# Establishing an early warning surveillance system in jails in Calabarzon, the Philippines, 2021

**DOI:** 10.5365/wpsar.2024.15.2.1083

**Published:** 2024-06-28

**Authors:** Karla May S Manahan, Alethea R De Guzman, Agnes B Segarra, Ma Nemia Sucaldito, Rammell Eric C Martinez

**Affiliations:** a Department of Health, Manila, Philippines.

## Abstract

The Philippines’ Republic Act 11332 (2020) mandates prisons, jails and detention centres to participate in disease surveillance, but currently no surveillance system exists in these facilities. This report aims to describe the piloting of an early warning disease surveillance system in 21 selected jails in Calabarzon from July to September 2021. Sites were selected based on congestion, proximity to health facilities and logistical capacity. Data sources, collection mechanisms and reporting tools were determined and health personnel were trained in the operation of the system. During the implementation period, the system detected 10 health events, with influenza-like illness and foodborne illness being the most common. Nine of these events were reported within 24 hours. The local health unit provided medications for clinical management and instructed jail nurses on infection prevention and control measures, including active case finding, the isolation of cases and the inspection of food handling. Twelve sites reported over 8 of the 10 weeks, with all sites reporting zero cases promptly. The challenges identified included insufficient workforce, slow internet speed and multitasking. It was concluded that the jail-based early warning surveillance system is feasible and functional, but the perceived benefits of jail management are crucial to the acceptability and ownership of the system. It is recommended to replicate the surveillance system in other penitentiaries nationwide.

Infectious diseases pose a significant global health concern. (*1*) Jail systems have particular challenges in controlling infectious diseases, as inmates are more vulnerable due to factors such as overcrowding and marginalized representation. (*2*-*4*) In the Philippines, jails were identified as potential hotspots for disease transmission when the COVID-19 pandemic struck the country. By March 2021, amidst the pandemic, 2416 detainees and 1295 personnel tested positive for COVID-19 within these facilities. Among those infected, 26 detainees and six personnel lost their lives to the virus (Bureau of Jail Management and Penology. Monthly health report on COVID-19, March 2021. Unpublished).

The Philippines’ Republic Act No. 11332 (2020), titled the “Mandatory Reporting of Notifiable Diseases and Health Events of Public Health Concern Act,” (*5*) mandates various public and private institutions to actively participate in disease surveillance and report cases of notifiable diseases. However, no disease surveillance system is currently established in Philippine jails, despite their dense and overcrowded populations heightening the risk of outbreaks, and limited disease reporting impeding early response. (*6*, *7*)

Disease surveillance enables continuous data collection and analysis to monitor disease burden, identify at-risk groups, track health outcomes and monitor targeted interventions. (*8*, *9*) Disease surveillance utilizes two key methods – indicator-based surveillance, which monitors predetermined health markers for routine analysis, and event-based surveillance, which captures unstructured data to detect emergent health events rapidly. Indicator-based surveillance follows a structured approach, whereas event-based surveillance offers flexibility in identifying unforeseen health risks. (*8*, *10*) An organized and rapid system for capturing epidemiological data is essential to detect and respond to public health threats promptly, ultimately reducing morbidity and mortality. (*11*)

To address the lack of disease surveillance in jails in the Philippines, a jail-based early warning surveillance system (JBEWS) was piloted in selected Calabarzon jails from July to September 2021. Calabarzon is an administrative region in central Luzon, Philippines, comprising five provinces and one highly urbanized city, representing around 15% of the Philippine population as of 2020. We describe the process of developing and establishing the disease surveillance system, which includes the assessment of the current reporting systems in the jails and the monitoring and evaluation of the implemented system.

## Methods

The development and implementation of the JBEWS in Calabarzon was conducted based on *A guide to establishing event-based surveillance* published by the World Health Organization (WHO). (*10*) The pilot study was conducted in three phases: the pre-implementation phase to design the surveillance system, the implementation of the system in the pilot sites, and the post-implementation review phase.

### Pilot sites

The study is conducted in jail units in the Philippines, which house unsentenced persons deprived of liberty or those undergoing trial; hence, the word “jail” is used throughout this article. The pilot study included 21 of the 62 jails in Calabarzon, representing 30% of facilities by detainee numbers. They were purposely sampled using criteria based on high congestion rates, proximity to external health facilities and the presence of nurses assigned to the jail. The jails were divided into three groups based on population size: Category A had 14 jails with over 500 detainees, Category B had 33 jails with 100–500 detainees, and Category C had 10 jails with fewer than 100 detainees. To ensure representation across facility types, 30% of the jails from each category were randomly selected. If a jail’s management opted not to participate in the study, another jail from the same category was randomly selected.

### Pre-implementation phase

The study team reviewed detainees’ health records from January 2020 to May 2021 and conducted key informant interviews and focus group discussions to inform the design of the surveillance system. A pilot facility assessment checklist was used to assess a facility’s readiness to establish a disease surveillance system, including the availability of a workforce, computers and reliable internet connectivity.

**Fig. 1:**
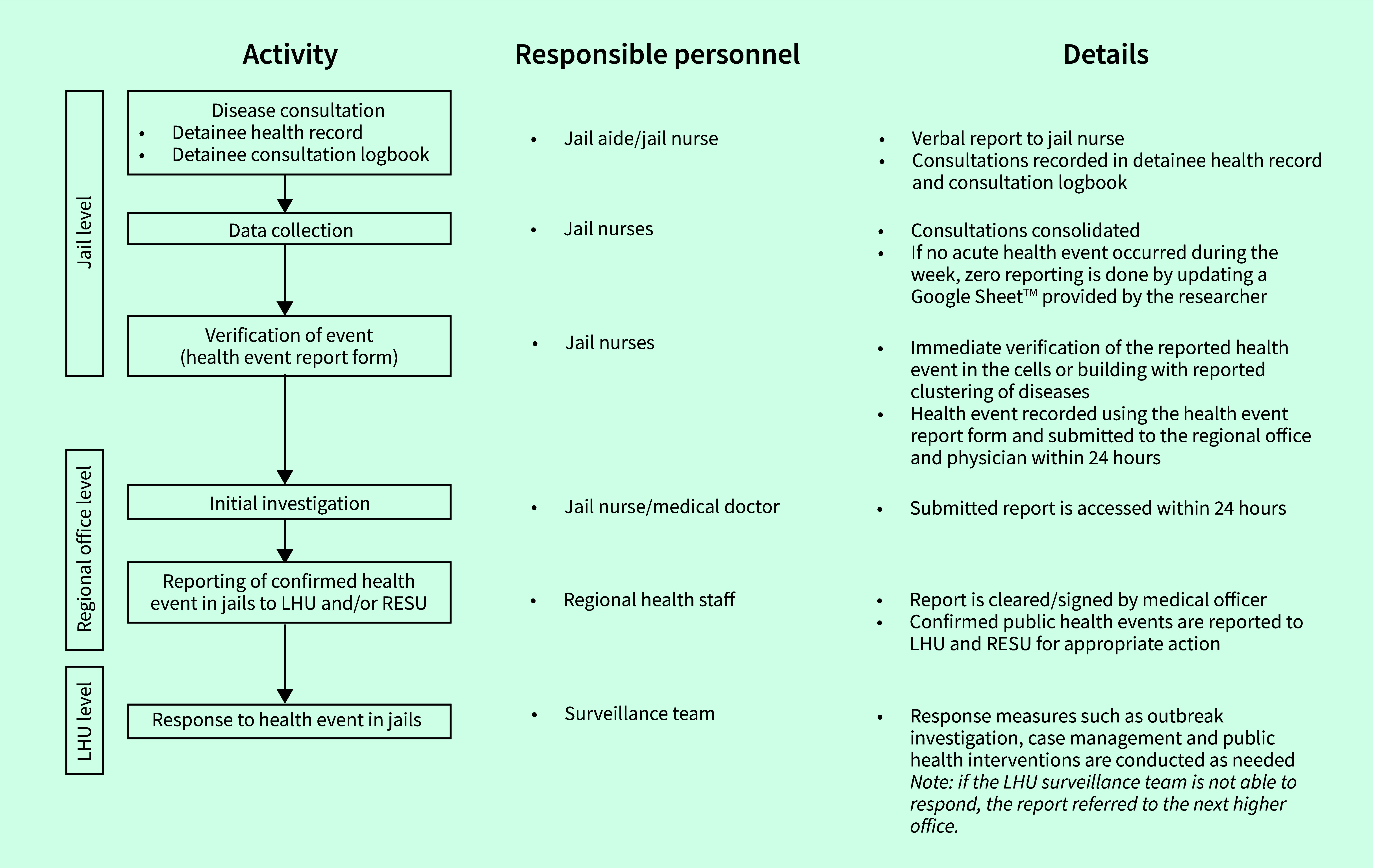
Design process flowchart for the jail-based early warning system, Calabarzon, the Philippines, from 1 July to 15 September 2021

The focus group discussion was facilitated by selected medical staff, including the jail nurses responsible for disease reporting and officials of the Bureau of Jail Management and Penology (BJMP) from the pilot sites. They discussed the project in terms of their experiences, beliefs, perceptions and attitudes in relation to the conduct of jail disease surveillance. A semi-structured questionnaire was applied to gather information on the perceived preparedness and needs for the establishment of the disease surveillance system. Interviews conducted by external partners in the disease surveillance network included questions on organizational structure, reporting methods, data management and response activities. The JBEWS was then designed based on the information collected (**Fig. 1**).

[**insert** Figure 1]

Prior to implementation of the JBEWS, a comprehensive 1-day training course was held for jail nursing staff, medical officers and disease surveillance officers from Calabarzon local health units. This training familiarized the participants with the operation of the JBEWS, including the data collection tools and reporting flow.

### Implementation phase

The implementation phase was conducted from 1 July to 15 September 2021, to give sufficient time for jail health events to be captured and to monitor consultations and hospitalizations among detainees and personnel. Data were then collected from the JBEWS on the events reported and responses. A submitted report was deemed timely if it was received within 24 hours of detecting the event. The completeness of reporting was assessed by the number of weekly consultation and hospitalization entries in the system. Data were analysed weekly using descriptive analyses and presented through tables and graphs in Microsoft Excel^®^. Electronic formats were stored securely, with the JBEWS team responsible for record keeping and archiving.

### Post-implementation phase

A focus group discussion with jail nurses was conducted to identify gaps and issues encountered during the implementation of the project. A final report on the outputs of the project was submitted to the BJMP and the Epidemiology Bureau of Calabarzon.

## Results

### Pre-implementation phase

The key informant interviews and focus group discussions provided a summary of how the existing jail health programme in Calabarzon operated. The BJMP in Calabarzon operates 62 jails accommodating 22 880 detainees with 1853 staff. The Health Service Division is responsible for implementing health programmes and generates a monthly health report obtained from the detainee health consultation logbook, which monitors consultations provided to detainees for 130 ailments. However, reporting compliance is low, except for tuberculosis (TB) and HIV. A high compliance rate with daily reporting of COVID-19 cases was also required.

The records review showed that from January 2020 to May 2021, 88 069 detainees underwent medical examinations, with the most common ailments being boils, upper respiratory tract infections and rashes (**Table 1**). There were no available records of medical consultations for jail personnel during the review.

**Table 1:** Top 10 reported conditions in the record review of jail detainees in Calabarzon, the Philippines, from January 2020 to May 2021

Condition	Number of consultations	Attack rate per 100 population
**Boils**	**10 959**	**48**
**Upper respiratory tract infection**	**10 891**	**47**
**Rash**	**5990**	**26**
**Hypertension**	**4865**	**21**
**Influenza**	**4168**	**18**
**Acute gastroenteritis**	**3896**	**17**
**Caries**	**3815**	**17**
**Migraine**	**3243**	**14**
**Infected wound**	**2596**	**11**
**Arthritis**	**2536**	**11**

*Source:* Bureau of Jail Management and Penology. Monthly health report on COVID-19, March 2021. Unpublished.

The pre-implementation assessment phase highlighted that few health events were reported through the existing system due to a shortage of health personnel, communication issues with the local health authorities, and a lack of communication protocols for detecting health events. In addition, jail staff reported that they lacked comprehensive disease surveillance training, and that the reporting system focused mainly on TB and HIV. They were also unfamiliar with Republic Act 11332 because it had not been disseminated to them. The Health Service Division acknowledged the importance of disease surveillance to prevent epidemics but reported that challenges arose due to the inadequate distribution of medical personnel, leading to multitasking among health-care professionals. Jail nurses recognized their role in reporting communicable diseases, but they were unaware of an existing early warning system and suggested capacity building and improved resources to enhance functionality. Limitations in reporting health events included a lack of coordination with local health units and unreliable internet connectivity.

### Description of the system

The JBEWS was designed based on the information gathered during the pre-implementation phase, the existing monthly reporting system and WHO’s *A guide to establishing event-based surveillance* (*10*) (**Fig. 1**).

When a jail nurse identified a disease cluster or an unusual surge in detainee consultations, they completed a health event form within 24 hours of detection. This form includes details such as time, location, number of cases detected, instances of mortality or hospitalization and responses. These reports were submitted to the decision-makers at the regional offices and the respective local health unit who implemented necessary measures such as outbreak investigation, case management and other public health interventions. The reports were also sent to the pilot study team. If there were no detected health events, the nurses were asked to submit a zero-health event report to the pilot study team at the end of each week in a Google Sheet^TM^.

The JBEWS is primarily an event-based surveillance system. However, zero case reporting was included, which is typically associated with indicator-based surveillance. The reason was to verify the absence of any unreported health events and ensure weekly reporting in the system.

### Implementation phase

The JBEWS captured 10 health events between 1 July and 15 September 2021. Two of the 21 jails each reported three events, while one reported two events and two reported one event – four influenza-like and three foodborne illnesses, an adverse event following immunization and conjunctivitis (**Table 2**). One case of pulmonary tuberculosis (PTB) was confirmed through laboratory testing and reported through the JBEWS (**Table 2**). The local health units promptly referred these health events to the regional doctor. Immediate responses included clinical management, the distribution of medications, active case finding and food-handling inspections to prevent further cases. The adverse event following immunization was also properly managed, and communicable diseases, such as influenza, conjunctivitis and PTB, were handled with active case finding and isolation to prevent further transmission (**Table 2**). All detected health events were between 2 and 10 cases, with no severe cases requiring hospitalization.

**Table 2:** Health event reported through the pilot jail-based early warning system in Calabarzon, the Philippines, from 1 July to 15 September 2021

Date of occurence	Pilot facility	Health event	No. of cases detected	Actions	Response
**5 July 2021**	**Jail A**	**Influenza**	**7**	**Verified and recorded**	**-LHU monitoring** **-Clinical management** **-Isolation of cases** **-Active case finding**
**9 July 2021**	**Jail B**	**Influenza**	**10**	**Verified and recorded**	**-LHU monitoring** **-Clinical management** **-Isolation of cases** **-Active case finding**
**13 July 2021**	**Jail A**	**Foodborne illness**	**4**	**Verified and recorded**	**-Clinical management** **-Active case finding** **-Inspection of food handling**
**21 July 2021**	**Jail B**	**Foodborne illness**	**2**	**Verified and recorded**	**-LHU monitoring** **-Clinical management** **-Active case finding** **-Inspection of food handling**
**21 July 2021**	**Jail C**	**Foodborne illness**	**2**	**Verified and recorded**	**-LHU monitoring** **-Clinical management** **-Active case finding** **-Inspection of food handling**
**29 July 2021**	**Jail C**	**Influenza**	**2**	**Verified and recorded**	**-LHU monitoring** **-Clinical management** **-Isolation of cases** **-Active case finding**
**6 August 2021**	**Jail D**	**Adverse effect following immunization**	**2**	**Verified and recorded**	**-LHU monitoring** **-Clinical management**
**3 September 2021**	**Jail B**	**Influenza**	**3**	**Verified and recorded**	**-LHU monitoring** **-Clinical management** **-Isolation of cases** **-Active case finding**
**5 September 2021**	**Jail A**	**Conjunctivitis**	**2**	**Verified and recorded**	**-Clinical management** **-Isolation of cases** **-Active case finding**
**5 September 2021**	**Jail E**	**Pulmonary tuberculosis**	**2**	**Verified and recorded**	**-LHU monitoring** **-Clinical management** **-Isolation of cases** **-Active case finding**

LHU: local health unit.

All reported health events were confirmed as genuine public heath events. Nine were reported to the JBEWS within 24 hours. All pilot sites reported zero cases in a timely manner. Twelve of the 21 pilot sites submitted weekly consultation and hospitalization entries at least eight times during the 10 weeks of the implementation phase.

### Post-implementation phase

The evaluation of the JBEWS identified challenges such as workforce shortages, slow internet connectivity and the need for multitasking, with the lack of personnel posing the most significant obstacle. The COVID-19 pandemic further disrupted health-service delivery. Suggestions on improving the system included increasing internet accessibility, augmenting the workforce, implementing a uniform daily consultation logbook and designating a focal person for each jail. Despite the challenges, the system proved effective in capturing, reporting and referring health events, leading to close monitoring and rapid case finding. It also strengthened collaboration between the jails and local health units. Jail nurses recommended implementing the system in other jails and regions to enhance disease control and outbreak prevention.

## Discussion

The JBEWS was successfully piloted in 21 jails in Calabarzon, the Philippines. The system detected health events with potential public health risks facilitating the timely assessment and response to control outbreaks. During the pilot study, the JBEWS was effective in capturing, verifying and reporting health events to higher authorities and local health units within 24 hours. The BJMP accepted the introduction of proactive measures through the implementation of a disease surveillance system to detect and respond to potential disease outbreaks within their facilities.

The integration of the existing reporting system into the JBEWS was a prudent approach for enhancing sustainability and efficiency, as it avoided the duplication of efforts. (*12*) The existing consultation logbook, which contained essential information to effectively capture health events, was a valuable resource for the new reporting system. In addition, positive perceptions and commitment from jail health personnel were crucial to its success, (*13*) and were highlighted as all pilot sites conducted zero case reporting during the 10 reporting weeks of the pre-implementation phase. The fact that only 11 of the 21 jails submitted timely consultation and hospitalization logbook entries indicates a need to strengthen personnel commitment to high-quality data reporting. (*14*)

The establishment of the JBEWS in more locations will require additional workforce, capacity building through training and improved internet connections. The use of other methods for 24/7 reporting that are crucial for event-based surveillance could also be explored, such as telephone calls, short message services (SMS), e-mails and faxes. (*15*) Raising awareness of the Philippines’ Republic Act 11332 for the mandatory reporting of infectious diseases among jail staff and enhancing coordination with local health units will facilitate data sharing and response. Addressing issues such as workforce shortages and slow internet connections when planning the improvement and sustainability of the JBEWS will enhance its effectiveness, provide a valuable tool to prevent and manage disease outbreaks in jails, and promote public health and safety. (*9*)

The pilot of the JBEWS encountered several limitations. As it was conducted during the peak of the COVID-19 pandemic, while jails were under lockdown, the usual service delivery mechanisms were restricted. The lockdown imposed stringent restrictions on movement, access and interactions within the jail setting, affecting the regular functioning of health-care services and surveillance procedures. This situation made it particularly challenging for external personnel, such as health-care professionals or surveillance officers, to enter the facility and conduct routine monitoring or investigations. Consequently, the surveillance methodology had to adapt to these limitations by using internal resources, such as jail nurses to facilitate active case finding and reporting within the confined environment. Despite these limitations, the primary goal of establishing an early warning system to detect outbreaks was achieved.

It is recommended that the BJMP should establish the JBEWS nationwide to enhance disease surveillance in the penal system. It is also recommended that the Philippines’ Republic Act 11332 for mandatory reporting be circulated to all offices and units within the BJMP. For the JBEWS to operate effectively, the nursing workforce would need to be increased and the internet connectivity improved. It is also suggested that the BJMP should create an interagency policy with the Department of Health to adopt the JBEWS, so that the Department of Health can support the implementation of the system and provide technical assistance for the public health response and laboratory services. By implementing these measures, the disease surveillance system in the jails can be strengthened, enabling timely reporting and response to potential health risks.

## References

[1] Michaud CM. Global burden of infectious diseases. In: Schaechter M, editor. Encyclopedia of Microbiology. 3rd ed. Academic Press; 2009. pp. 444–54., 10.1016/B978-012373944-5.00185-1

[2] Preventing infectious diseases in prisons: a public health and human rights imperative. London: Penal Reform International; 2015. Available from: https://www.penalreform.org/blog/preventing-infectious-diseases-in-prisons-a-public-health/, accessed 29 June 2020.

[3] Ndeffo-Mbah ML, Vigliotti VS, Skrip LA, Dolan K, Galvani AP. Dynamic models of infectious disease transmission in prisons and the general population. Epidemiol Rev. 2018 Jun 1;40(1):40–57. 10.1093/epirev/mxx014 29566137 PMC5982711

[4] Nijhawan AE. Infectious diseases and the criminal justice system: a public health perspective. Am J Med Sci. 2016 Oct;352(4):399–407. 10.1016/j.amjms.2016.05.020 27776722 PMC5119815

[5] Mandatory Reporting of Notifiable Diseases and Health Events of Public Health Concern Act. 2020. Republic Act No. 11332. Available from: https://ntp.doh.gov.ph/download/mandatory-reporting-of-notifiable-diseases-and-health-events-of-public-health-concern-act/, accessed 3 April 2024.

[6] Yarcia LE. Kalusugan sa kulungan: examining the policy for people living with HIV/AIDS and hepatitis C in Philippine prisons. London: International Drug Policy Consortium; 2018. Available from: https://idpc.net/publications/2018/05/kalusugan-sa-kulungan-examining-the-policy-for-people-living-with-hiv-aids-and-hepatitis-c-in-philippine-prisons, accessed 20 January 2021.

[7] Morales NJ. Jails, justice system at breaking point as Philippine drugs war intensifies. Reuters. 1 September 2017. Available from: https://www.reuters.com/article/us-philippines-justice/jails-justice-system-at-breaking-point-as-philippine-drugs-war-intensifies-idUSKCN1BB39F, accessed 20 January 2021.

[8] Event-based surveillance. Atlanta (GA): United States Centers for Disease Control and Prevention; 2023. Available from: https://www.cdc.gov/globalhealth/healthprotection/gddopscenter/event-based-surveillance.html, accessed 15 September 2023.

[9] Kuehne A, Keating P, Polonsky J, Haskew C, Schenkel K, Le Polain de Waroux O, et al. Event-based surveillance at health facility and community level in low-income and middle-income countries: a systematic review. BMJ Glob Health. 2019 Dec 10;4(6):e001878. 10.1136/bmjgh-2019-001878 31908863 PMC6936563

[10] A guide to establishing event-based surveillance. Manila: WHO Regional Office for the Western Pacific; 2008. Available from: https://iris.who.int/handle/10665/207737, accessed 14 December 2021.

[11] Flanigan TP, Zaller N, Taylor L, Beckwith C, Kuester L, Rich J, et al. HIV and infectious disease care in jails and prisons: breaking down the walls with the help of academic medicine. Trans Am Clin Climatol Assoc. 2009;120:73–83. 19768164 PMC2744543

[12] Bawa SB, Olumide EA, Umar US. The knowledge, attitude and practices of the reporting of notifiable diseases among health workers in Yobe State, Nigeria. Afr J Med Med Sci. 2003 Mar;32(1):49–53. 15030066

[13] Bautista MB. 31 integrated jail management system for the Bureau of Corrections. 2014. Available from: https://api.semanticscholar.org/CorpusID:173983655, accessed 13 February 2021.

[14] Centers for Disease Control (CDC). Guidelines for evaluating surveillance systems. MMWR Suppl. 1988 May 6;37(5):1–18. 3131659

[15] Usman R, Bakare L, Akpan UO, Dalhat M, Dada AO, Okudo I, et al. Establishing event-based surveillance system in Nigeria: a complementary information generating platform for improved public health performance, 2016. Pan Afr Med J. 2022 May 24;42:63. 10.11604/pamj.2022.42.63.29621 35949466 PMC9338694

